# Beyond Imaging: A 12-Year Clinicopathological Analysis of Surgically Excised Neurocysticercosis

**DOI:** 10.7759/cureus.111697

**Published:** 2026-06-29

**Authors:** Nandita Chaudhary, Neha Nigam, Rahul K Gupta, Sushila Jaiswal, Ritu Verma, Ashutosh Kumar, Awadhesh Jaiswal, Sumit Sachan, Anuradha Singh

**Affiliations:** 1 Department of Pathology, Sanjay Gandhi Postgraduate Institute of Medical Sciences, Lucknow, IND; 2 Department of Neurosurgery, Sanjay Gandhi Postgraduate Institute of Medical Sciences, Lucknow, IND; 3 Department of Anesthesiology, Sanjay Gandhi Postgraduate Institute of Medical Sciences, Lucknow, IND; 4 Department of Radiology, Sanjay Gandhi Postgraduate Institute of Medical Sciences, Lucknow, IND

**Keywords:** central nervous system, histopathology, intracranial cystic lesion, neurocysticercosis, parasitic cysts, taenia solium

## Abstract

Background: Neurocysticercosis (NCC), caused by the larval stage of *Taenia solium*, is the most prevalent parasitic infection of the central nervous system (CNS) and a leading cause of acquired epilepsy in endemic regions. Although neuroimaging has substantially improved the diagnosis of NCC, radiological appearances may vary according to the stage of the parasite and often overlap with neoplastic, inflammatory, and other infectious lesions. Consequently, histopathological examination remains the definitive diagnostic modality in surgically excised cases.

Methods: This retrospective clinicopathological study included 26 histopathologically diagnosed cases of surgically excised NCC over a 12-year period from January 2014 to December 2025 at a tertiary care center in Northern India. Demographic profile, clinical presentation, and radiological findings were retrieved from patient case files and the hospital information system. Operative notes were reviewed to document intraoperative findings and the provisional intraoperative diagnosis. Histological sections were reviewed to confirm the diagnosis, evaluate parasite morphology, and assess host inflammatory response and associated tissue changes. The diagnostic accuracy of radiological tests and intraoperative assessment was evaluated, with histopathological diagnosis as the gold standard.

Results: Twenty-six patients were identified, ranging in age from 11 to 68 years (mean, 32.0 years), with a male predominance (male: female ratio, 2.7:1). The majority of cases presented with signs and symptoms of raised intracranial pressure, including headache (22/26, 84.6%), followed by vomiting (16/26, 61.5%). Obstructive hydrocephalus was noted in 73.1% (19/26) of cases, all of which were localized in ventricles or cisterns. Depending on the location of the parasitic cyst, other presenting manifestations included focal or generalized seizures (6/26, 23.1%), visual disturbances (3/26, 11.5%), gait abnormalities (3/26, 11.5%), meningitis (3/26, 11.5%), and focal neurological deficits (2/26, 7.7%). The mean duration of symptoms was 8.2 months (range, 1-36 months). Lesion size ranged from 8 to 60 mm (mean, 21.3 mm). Preoperative radiological diagnosis correctly suggested NCC in 53.8% (14/26) of cases, whereas intraoperative diagnosis was accurate in 69.2% (18/26). Histopathological examination established the diagnosis in all cases and demonstrated variable stages of parasite degeneration, with an associated inflammatory reaction in 30.8% (8/26) of cases and calcification in 19.2% (5/26) of cases. The most common morphological stages of the parasite were the vesicular and colloidal stages, together accounting for 61.5% (16/26) of cases.

Conclusions: CNS NCC exhibits diverse clinical and radiological manifestations that may pose significant diagnostic challenges. In surgically excised lesions, histopathological evaluation remains indispensable for definitive diagnosis and provides valuable insights into parasite viability, host inflammatory response, and disease evolution. Our findings underscore the limitations of imaging alone and highlight the complementary role of histopathology in achieving accurate diagnosis and guiding appropriate clinical management.

## Introduction

Neurocysticercosis (NCC), caused by infection with the larval stage of *Taenia solium*, is the most common parasitic disease of the central nervous system (CNS) and remains a major public health problem in many low- and middle-income countries, particularly in Asia, Latin America, and sub-Saharan Africa, as quoted by Hisaeda [1] and Garcia et al. [2]. Owing to increased global travel and migration, NCC is also being increasingly recognized in nonendemic regions, making it a disease of worldwide clinical significance [2]. The clinical manifestations of NCC are highly variable and depend on the number, size, location, viability, and stage of degeneration of the cysticerci, as well as the host inflammatory response [3]. Patients commonly present with seizures, headache, focal neurological deficits, hydrocephalus, or features of raised intracranial pressure, often mimicking other infectious, inflammatory, or neoplastic CNS lesions [2,3].

Advances in neuroimaging, particularly computed tomography (CT) and magnetic resonance imaging (MRI), have considerably improved the diagnosis of NCC. Characteristic imaging findings, including cystic lesions with an eccentric scolex, ring-enhancing lesions, and calcified nodules, frequently allow a presumptive diagnosis [4]. However, radiological appearances may vary according to the evolutionary stage of the parasite and can overlap with those of tuberculomas, abscesses, gliomas, metastases, and other space-occupying lesions of the CNS. Consequently, a definitive preoperative diagnosis is not always possible, especially in surgically excised lesions presenting with atypical radiological features, as shown by Del Brutto et al. [4] and Del Brutto [5].

Histopathological examination remains the gold standard for confirming the diagnosis of NCC and provides valuable information regarding parasite morphology, stage of degeneration, associated inflammatory response, and secondary tissue changes such as fibrosis and calcification [1,5]. Furthermore, histopathological evaluation offers important insights into the pathogenesis and natural evolution of the disease, complementing clinical and radiological findings [5].

Despite the high prevalence of NCC in endemic regions, detailed clinicopathological studies of surgically excised CNS lesions remain scarce. Accordingly, the primary objective of the present study was to characterize the clinicopathological spectrum of histopathologically confirmed NCC over a 12-year period. The secondary objective was to determine the diagnostic accuracy of radiological and intraoperative assessments in relation to the final histopathological diagnosis, thereby highlighting the diagnostic value of histopathological examination in surgically managed cases.

## Materials and methods

Study design and setting

This retrospective clinicopathological study included 26 histopathologically diagnosed cases of NCC surgically excised under general anesthesia over a 12-year period from January 2014 to December 2025 at a tertiary care center in Northern India. The study utilized anonymized patient data, with strict maintenance of confidentiality and adherence to institutional ethical standards.

Inclusion and exclusion criteria

The study included all surgically excised intracranial and intraspinal lesions with a definitive histopathological diagnosis of NCC during the study period, for which adequate tissue sections, clinical details, radiological findings, and operative records were available. Cases suspected solely on the basis of clinical or radiological findings without tissue confirmation, specimens with inadequate tissue for review, cases with incomplete clinicoradiological data, and cysticercosis involving sites outside the CNS were excluded from the study.

Data collection

Clinical and radiological records were retrieved from the patient case files and the hospital information system. Demographic and clinical variables, including age, gender, presenting symptoms, duration of symptoms, and neurological manifestations, were recorded. Radiological findings obtained from CT and/or MRI reports were reviewed, including lesion location, size, and radiological diagnosis. Operative notes were examined to document intraoperative findings and the provisional diagnosis rendered by the neurosurgical team. The diagnostic accuracy of radiological tests and intraoperative assessment was evaluated, with histopathological diagnosis as the gold standard and the final correct diagnosis.

Histopathological evaluation

All available hematoxylin and eosin-stained slides were retrieved and reviewed by a single experienced pathologist. Cases with diagnostic uncertainty were reviewed jointly with a senior pathologist, and a consensus diagnosis was reached. Histopathological examination focused on the identification of characteristic parasitic structures, including the cyst wall, scolex, suckers, and remnants of the larval body for confirmation of diagnosis. The presence and extent of associated host tissue responses, including inflammatory reaction, necrosis, calcification, and reactive gliosis, were analyzed. The stage of NCC was determined histopathologically based on the morphological preservation of the parasite and the associated host tissue response. Lesions were classified into four stages: 1) vesicular stage, characterized by a viable cyst with a thin translucent cyst wall and absent or minimal surrounding inflammatory reaction; 2) vesicular-colloidal stage, showing degeneration of the parasite associated with turbid cyst contents and onset of mild inflammatory response; 3) granular nodular stage, characterized by further degeneration of the parasite with collapse of the cyst cavity and prominent inflammatory and reparative changes; and 4) nodular calcified stage, demonstrating a nonviable, retracted, and calcified parasite as described by Dametto [3].

Statistical analysis

Statistical analysis was performed using Microsoft Excel (Microsoft Corporation, Redmond, WA). Descriptive statistics were used to summarize the study data. Continuous variables such as age, duration of symptoms, and lesion size were expressed as mean values and ranges. Categorical variables, including sex distribution, clinical manifestations, radiological diagnoses, and histopathological features, were summarized as frequencies and percentages.

## Results

Demographic and clinical characteristics

A total of 26 histopathologically diagnosed cases of NCC were identified during the 12-year study period. The patients ranged in age from 11 to 68 years, with a mean age of 32.0 years. The majority of patients presented in the second to fourth decades of life. There was male predominance, with 19 males and seven females, yielding a male-to-female ratio of 2.7:1 (Table 1).

**Table 1:** Neurocysticercosis: patient age and gender distribution, lesion size, and duration of presenting symptoms (N = 26) M:F ratio = 2.7:1

Variables	Range	Mean
Age (years)	11-68	32.0
Size of lesion (mm)	8-60	21.3
Duration of symptoms (months)	1-36	8.2

The duration of symptoms ranged from 1 to 36 months, with a mean duration of 8.2 months (Table 1). The majority of cases presented with signs and symptoms of raised intracranial pressure, including headache (22/26, 84.6%), which was the most common symptom, followed by vomiting (16/26, 61.5%). Obstructive hydrocephalus was noted in 73.1% (19/26) of cases, all of which were localized in ventricles or cisterns. Depending on the location of the parasitic cyst, other presenting manifestations included focal or generalized seizures (6/26, 23.1%), visual disturbances (3/26, 11.5%), gait abnormalities (3/26, 11.5%), meningitis like presentation with fever and neck rigidity (3/26, 11.5%), and focal neurological deficits like spastic quadriparesis, limb weakness, etc. (2/26, 7.7%). One patient presented with altered sensorium with features of pontine and extrapontine myelinolysis (Table 2).

**Table 2:** Clinical characteristics and site distribution of NCC (N = 26) ^#^Parenchymal cases include six intracranial and one intramedullary spinal NCC ^##^Intraventricular cases include seven fourth ventricular and four third ventricular NCC ^###^Cisterns include two prepontine, two quadrigeminal, and one cisterna magna NCC NCC: neurocysticercosis

Variables	Number of cases	Percentage (%)
Presenting sign and symptoms		
Headache	22	84.6
Vomiting	16	61.5
Hydrocephalus	19	73.1
Seizures	6	23.1
Gait abnormalities	3	11.5
Visual disturbances	3	11.5
Meningitis-like presentation	3	11.5
Neurological deficits	2	7.7
Altered sensorium with pontine myelinolysis	1	3.8
Site		
Parenchymal^#^	7	26.9
Intraventricular^##^	11	42.3
Cisterns^###^	5	19.2
Left sylvian fissure	1	3.8
Septum pellucidum	1	3.8
Left parasellar region	1	3.8

Anatomical distribution

The ventricular system was the most frequently involved site, observed in 11 of 26 cases (42.3%), including seven fourth-ventricular and four third-ventricular lesions. Parenchymal involvement was noted in seven cases (26.9%), comprising six intracranial and one intramedullary spinal NCC. Cisternal lesions accounted for five cases (19.2%), including two prepontine, two quadrigeminal cistern, and one cisterna magna lesion. Isolated involvement of the left sylvian fissure, septum pellucidum, and left parasellar region was observed in one case (3.8%) each (Table 2). This distribution highlights the predominance of ventricular and cerebrospinal fluid space involvement in the surgically treated cohort.

Radiological and intraoperative findings

**Figure 1:**
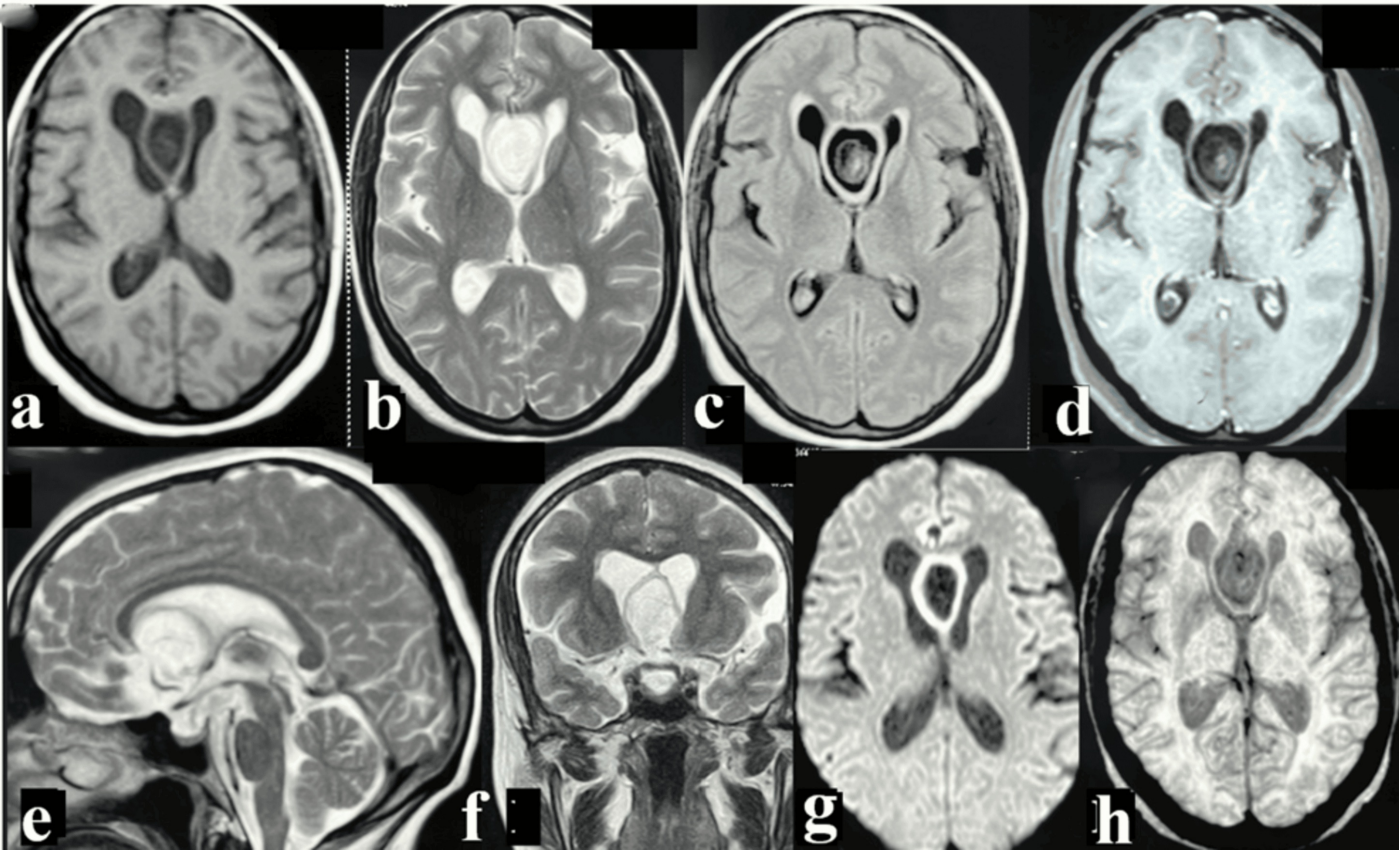
(a) T1-weighted MRI axial section showing a single well-defined cystic lesion in the midline at the level of the frontal horn of the lateral ventricle. Within the lesion, there were isointense and hyperintense signal changes. (b) On T2-weighted images, the lesion was hyperintense, and no surrounding edema was noted. (c) There was complete suppression of signals within the lesion on the T2 flair sequence. (d) The contrast enhancement was minimal, with only small enhancing patches along the rim of the lesion. (e,f) The lesion was located within the septum pellucidum in the anterior part (frontal horn) of the lateral ventricle. (g) The rim of the lesion showed diffusion restriction. (h) No intralesional blooming was seen within the lesion MRI: magnetic resonance imaging

Neuroimaging revealed CNS lesions of variable size and morphology. Lesion size ranged from 8 to 60 mm, with a mean diameter of 21.3 mm (Table 1). Neurocysticercosis commonly appeared as a cystic lesion (Figure 1) with or without scolex (cyst with mural nodule), ring-enhancing lesion, or calcified lesions on MRI. Intraventricular NCC appeared as intraventricular cysts and was commonly associated with upstream obstructive hydrocephalus.

A preoperative radiological diagnosis of NCC was suggested in 14 of 26 cases (53.8%), whereas the remaining 12 lesions were radiologically interpreted as alternative pathologies, including other parasitic cysts (four cases as hydatid cysts), neoplastic (one ependymoma and one low-grade glioma), noninfective cyst (one arachnoid cyst), and unclassified lesions (five cases) (Table 3). Three of the four lesions radiologically diagnosed as hydatid cysts were subsequently confirmed on histopathological examination to represent the racemose variant of NCC. On neuroimaging, these lesions appeared as multiloculated, thin-walled cystic masses within the ventricular system, lacking an identifiable scolex and exhibiting a characteristic “bunch-of-grapes” configuration. The lesions were associated with minimal perilesional edema, absence of significant contrast enhancement, and varying degrees of mass effect resulting in obstructive hydrocephalus. As these imaging features are common to both intracranial hydatid cysts and racemose NCC, accurate preoperative differentiation proved challenging. Histopathological evaluation was, therefore, crucial in establishing the definitive diagnosis by demonstrating the characteristic morphological features of racemose NCC.

**Table 3:** Diagnostic accuracy of radiological and intraoperative assessment (N = 26)

Diagnostic modality	Correct diagnosis, n/N	Accuracy (%)
Radiological diagnosis	14/26	53.8
Intraoperative impression	18/26	69.2
Histopathological diagnosis	26/26	100

Intraoperatively, NCC was suspected in 18 of 26 cases (69.2%). In the remaining eight cases, intraoperative impression was inaccurate (five suspected cases of hydatid cyst, one ependymoma, one pilocytic astrocytoma, and one inconclusive), and the definitive diagnosis was established only after histopathological examination (Table 3).

Histopathological findings

**Figure 2:**
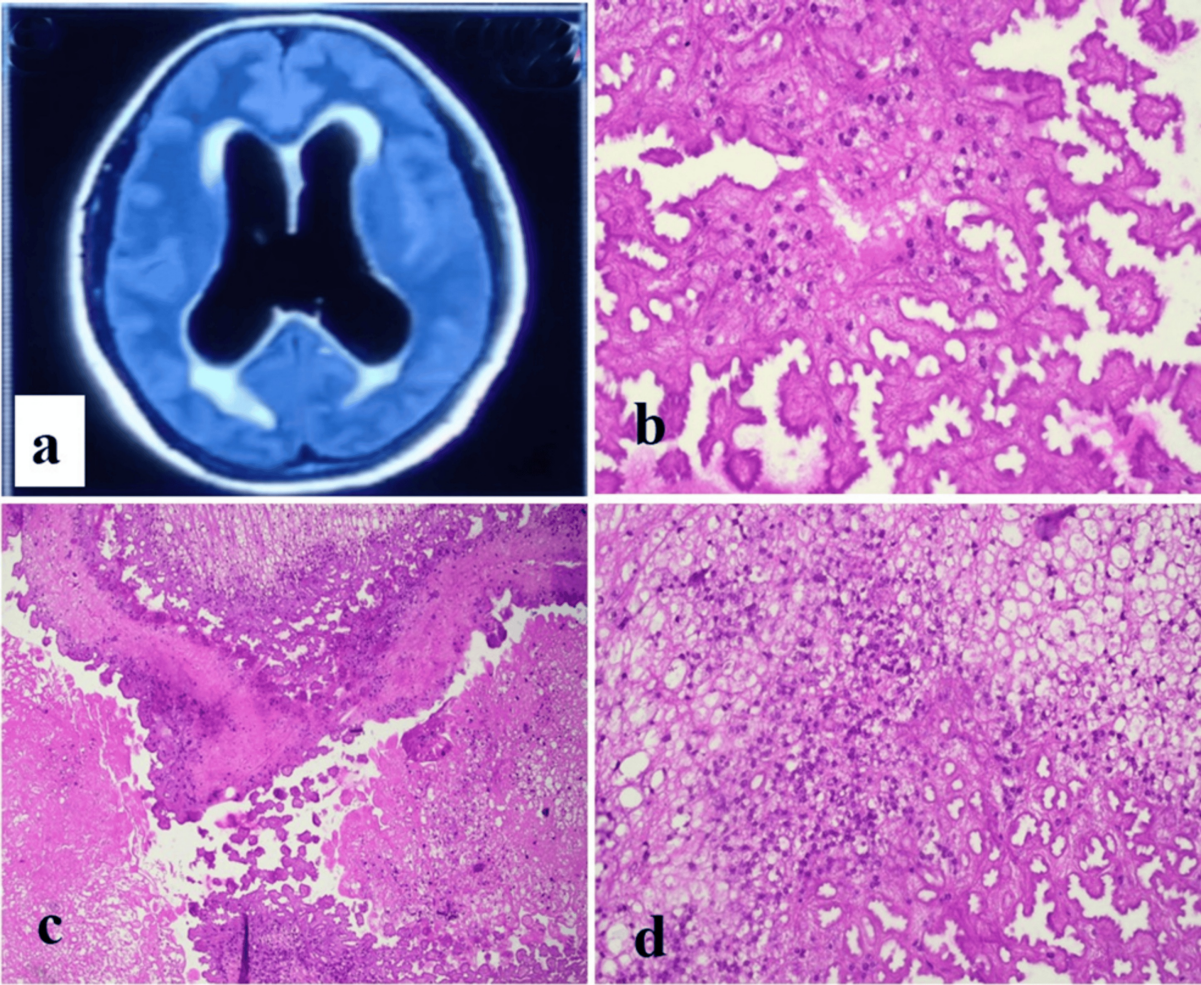
Fourth ventricular neurocysticercosis. (a) Hydrocephalus with periventricular ooze on radiology. (b) H&E (200×): neurocysticercosis cyst wall with cobblestone appearance. (c) H&E (200×): cyst wall with necrosis (left lower corner). (d) H&E (200×): inflammatory reaction adjacent to cyst wall H&E: hematoxylin and eosin

**Figure 3:**
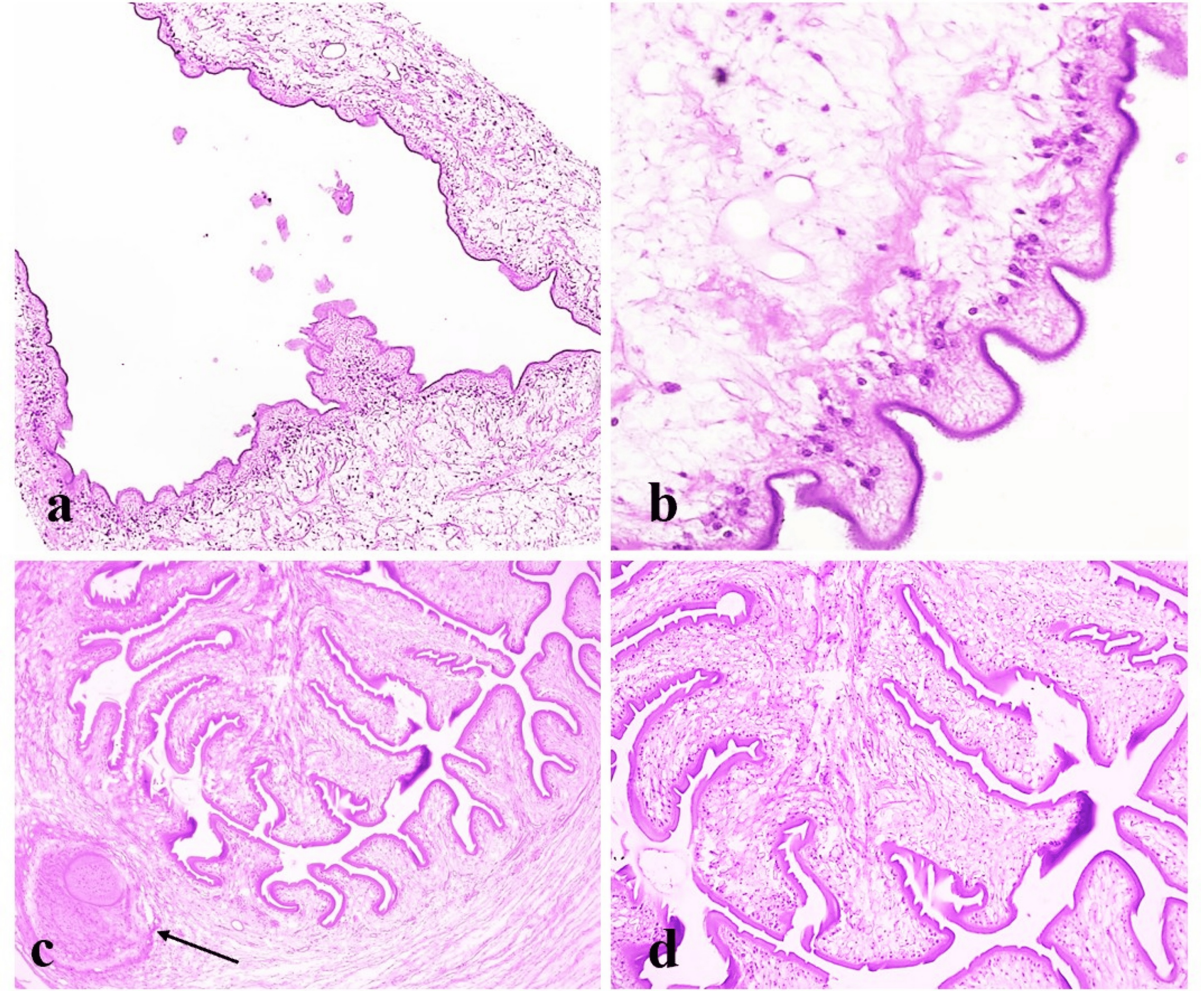
Hematoxylin and Eosin staining: (a) 100× and (b) 400×. Photomicrographs of the neurocysticercosis cyst wall composed of outer cuticular, middle nuclear, and inner reticular layer, (c) 200×, complete scolex composed of folded parenchyma with sucker (black arrow) and (d) 400×, folded parenchyma of parasite

Histopathological examination confirmed NCC in all cases. The lesions demonstrated varying stages of parasite degeneration and host tissue response. Preserved parasitic structures were composed of a characteristic eosinophilic multilayered cyst wall composed of an outer cuticle layer, a middle nuclear layer, and an inner parenchymal or reticular layer, diagnostic of cysticercosis. The cyst wall commonly showed a cobblestone appearance (Figure 2). A few of the cases showed a complete scolex with folded parenchyma and suckers (Figure 3). The most common morphological stage of the parasite was the vesicular and colloidal stage, together accounting for 61.5% (16/26) of cases.

A significant inflammatory response was present in eight cases (30.8%). The inflammatory infiltrate comprised lymphocytes, plasma cells, histiocytes, eosinophils, and occasional multinucleated giant cells. Two cases (7.7%) showed necrosis and granular debris (Figure 2). Reactive gliosis in the adjacent neural tissue was observed in two out of three cases containing glial parenchyma. Calcification, indicative of chronic or involuting lesions, was identified in 19.2% of cases (Table 4).

**Table 4:** Histopathological characteristics of neurocysticercosis (N = 26)

Morphological features	Number of cases, n/N	Percentage (%)
Cysticercal cyst (viable to degenerated)	26/26	100
Inflammatory reaction	8/26	30.8
Calcification	5/26	19.2
Normal glial parenchyma	3/26	11.5
Necrosis	2/26	7.7
Vesicular stage	4/26	15.4
Vesicular colloidal stage	12/26	46.2
Granular nodular stage	8/26	30.7
Nodular calcified stage	02/26	7.7

## Discussion

NCC remains the most common parasitic infection of the CNS and a major cause of neurological morbidity in endemic regions [1,2]. Despite advances in neuroimaging and serological testing, diagnosis may be challenging because the clinical and radiological manifestations vary with the anatomical location, the stage of parasite degeneration, and the host immune response [4,5]. The present study provides a clinicopathological analysis of 26 surgically excised and histopathologically confirmed NCC cases, highlighting the continuing importance of tissue diagnosis in lesions with atypical presentations or uncertain radiological interpretations.

The demographic profile of our cohort is consistent with previous studies reporting a predominance of NCC in young and middle-aged adults [5-8]. The mean age of 32 years closely parallels that reported by Milenković et al. [8] in their systematic review of ventricular NCC (33.1 years). A male predominance was also observed, a finding frequently reported in studies from endemic regions [5,9,10]. The systematic review on spinal NCC by Garg et al. [10] also showed similar results, with a male predominance (62.4% male patients, with an M:F ratio of 1.7:1) and a mean age at diagnosis of 39.5 years. Although the underlying reasons remain incompletely understood, the gender predilection is likely related to environmental, dietary, socioeconomic, and occupational exposure factors rather than biological susceptibility. Thus, our findings align with the established epidemiological profile of NCC in endemic regions.

An important observation in the present study was the predominance of extraparenchymal disease. Intraventricular lesions accounted for 42.3% of cases, followed by cisternal lesions (19.2%), whereas parenchymal lesions constituted only 26.9%. This distribution differs from population-based studies in which parenchymal NCC is the most common form of disease and is typically associated with seizure disorders [2,5]. However, it is comparable to surgically managed cohorts, where ventricular and cisternal cysticerci are overrepresented because of their tendency to cause obstructive hydrocephalus, raised intracranial pressure, and mass effect requiring neurosurgical intervention. Kumar et al. [9] reported that intraventricular cysticerci are an important cause of hydrocephalus in children requiring operative management, and Sharma et al. [11] reported that intraventricular NCC occurs in approximately 7%-33% of cases, with the fourth ventricle being the most frequent location. Similarly, seven of the 11 ventricular lesions in our series were located in the fourth ventricle, supporting previous observations regarding the predilection of cysticerci for this extraparenchymal site [9,11,12]. The higher proportion of extraparenchymal lesions observed in our cohort is likely attributable to selection bias inherent to the study design, which was restricted to surgically resected cases. In contrast, parenchymal NCC is frequently diagnosed radiologically and managed medically, reducing the likelihood of tissue sampling and histopathological confirmation.

The predominance of ventricular and cisternal lesions was reflected in the clinical presentation of our patients. Headache, vomiting, and hydrocephalus were the most common manifestations, whereas seizures were relatively infrequent. These findings contrast with the classical presentation of parenchymal NCC, in which seizures are the predominant symptom [2,5,6], owing to the predominance of parenchymal disease as seen in the study done by Bhatt et al. [6], which showed predominant parenchymal location (47.6% cases) followed by intraventricular lodgment of parasite. However, they closely resemble observations from studies of ventricular disease. Milenković et al. [8], in a systematic review of 48 cases of lateral ventricular NCC, reported headache in 87.8%, vomiting in 51.2%, and hydrocephalus in 81.3% of patients with ventricular NCC, findings remarkably similar to those observed in our cohort. Likewise, Alam et al. [13] described patients with NCC presenting primarily with headache in the absence of seizures. Collectively, these observations underscore the critical influence of lesion location on clinical manifestations and disease course. Thus, our study expands upon existing literature by demonstrating that surgically excised NCC represents a distinct clinical subset characterized predominantly by cerebrospinal fluid pathway obstruction rather than epileptic manifestations.

Cisternal lesions accounted for 19.2% of cases in the present study and were predominantly located within the prepontine and quadrigeminal cisterns. These locations have been recognized as common sites of subarachnoid NCC because of their close relationship with cerebrospinal fluid pathways [5,14]. Extraparenchymal NCC involving basal cisterns is often associated with arachnoiditis, chronic inflammation, and cerebrospinal fluid obstruction, thereby contributing to increased intracranial pressure and hydrocephalus [5]. The findings of our study are therefore in agreement with previous radiologic-pathologic investigations by Kimura-Hayama et al. [14], which emphasized the distinct clinicopathological behavior of cisternal NCC compared with parenchymal disease.

Parenchymal NCC accounted for only seven cases in our series, including six intracranial lesions and one intramedullary spinal lesion. Although spinal involvement is uncommon, its occurrence in our cohort is noteworthy. Previous studies have estimated that spinal NCC constitutes only 1.5%-3% of all NCC cases, with most lesions occurring in the subarachnoid compartment and intramedullary involvement being particularly rare [15]. Recent systematic reviews by Garg et al. [10] have similarly emphasized the rarity of spinal disease and the limited number of reported cases worldwide. Consequently, the identification of an intramedullary spinal NCC case in our study expands upon existing literature and highlights the broad anatomical spectrum that may be encountered in histopathologically confirmed NCC.

The presence of isolated lesions in uncommon locations, including the sylvian fissure, septum pellucidum, and parasellar region, further illustrates the remarkable anatomical diversity of NCC. Such atypical locations may pose significant diagnostic challenges because their radiological appearance often overlaps with neoplastic, inflammatory, or other parasitic lesions [4,14]. These findings support the observations of Del Brutto et al. [4] and Kimura-Hayama et al. [14], who emphasized that lesion location and stage of degeneration substantially influence radiological interpretation and diagnostic certainty.

Overall, the anatomical distribution observed in the present study demonstrates a predominance of surgically significant extraparenchymal disease and provides important clinicopathological insights that complement existing literature. While the findings differ from population-based studies in which parenchymal NCC predominates, they align closely with reports focusing on surgically managed ventricular and cisternal disease. Furthermore, the inclusion of rare spinal and atypically located lesions expands current knowledge regarding the pathological spectrum of NCC and reinforces the importance of histopathological examination in establishing a definitive diagnosis across diverse anatomical locations [6,8,14,15].

Neuroimaging remains the cornerstone of NCC diagnosis and forms the basis of the revised diagnostic criteria proposed by Del Brutto et al. [4]. Classical radiological features, including a cyst containing an eccentric scolex, ring-enhancing lesions, and calcified nodules, often permit confident diagnosis. Nevertheless, radiological appearances vary considerably across the evolutionary stages of the parasite and may overlap with neoplastic, inflammatory, or other infectious lesions [5,14]. In our study, radiological diagnosis correctly identified NCC in only slightly more than half of the cases (53.8%), reflecting the challenges posed by atypical lesion locations and varying stages of degeneration. This diagnostic accuracy is lower than that reported in studies involving typical parenchymal lesions [4,5], but is comparable to observations from surgically excised and atypically located lesions where radiological overlap poses substantial diagnostic challenges. Similar diagnostic limitations have been highlighted by Kimura-Hayama et al. [14], who demonstrated the broad spectrum of radiologic-pathologic correlations encountered in NCC. Our findings, therefore, expand upon previous studies by quantitatively demonstrating the limitations of imaging in surgically selected NCC cases and reinforce the concept that atypical lesions frequently remain diagnostically ambiguous until histological evaluation is performed.

Interestingly, intraoperative diagnostic accuracy (69.2%) was only modestly higher than preoperative radiological accuracy. Similar discrepancies between clinical, radiological, and operative impressions have been noted by Del Brutto et al. [4], who emphasized that definitive diagnosis may remain elusive in lesions lacking characteristic imaging features. The present findings align with these observations and further demonstrate that even direct surgical visualization may not reliably distinguish NCC from other cystic CNS lesions. Consequently, histopathological examination remains indispensable for diagnostic confirmation.

Histopathological examination established the diagnosis in all cases and provided additional information regarding parasite viability, stage of degeneration, and host tissue response. Dametto [3] emphasized that histopathological assessment offers unique insights into parasite-host interactions that cannot be adequately evaluated through imaging alone. Our findings strongly support this assertion. The characteristic laminated eosinophilic cyst wall and residual parasitic structures were readily identified in viable and partially degenerated lesions, permitting definitive diagnosis even in cases where radiological and operative findings were inconclusive. Thus, our study aligns with previous pathological investigations while reinforcing the central role of histopathology in NCC diagnosis.

The host inflammatory response observed in the present series showed considerable variation, ranging from minimal chronic inflammation to dense mixed inflammatory infiltrates composed of lymphocytes, plasma cells, histiocytes, eosinophils, and multinucleated giant cells. Similar histological patterns were described by Dametto [3], who demonstrated that inflammation intensifies as cysticerci undergo degeneration and release antigenic material into surrounding tissues. Our findings are therefore in agreement with prior studies and further support the concept that clinical manifestations are primarily mediated by the host immune response rather than by the physical presence of the parasite itself. Moreover, by correlating inflammatory changes with varying stages of degeneration, the present study expands upon earlier observations and illustrates the dynamic pathological spectrum encountered in surgically excised lesions.

Calcification was identified in a subset of cases (19.2%) and was generally associated with degenerated or nonviable parasites. Traditionally regarded as an inactive end-stage lesion, calcified NCC is increasingly recognized as a potential source of persistent inflammation and recurrent neurological symptoms. Nash et al. [16] demonstrated that calcified lesions may be associated with perilesional edema and ongoing histological inflammation, indicating that parasite death does not necessarily signify complete biological inactivity. Our observations support this evolving understanding and highlight the value of histopathological assessment in accurately determining disease stage.

One of the most significant contributions of the present study is the demonstration of the complementary relationship between neuroimaging and histopathology. While imaging remains essential for lesion detection, localization, and surgical planning, histopathology provides direct evidence of parasite morphology, viability, degeneration, inflammation, gliosis, fibrosis, and calcification. This observation extends the conclusions of Del Brutto et al. [4], who emphasized the importance of integrating clinical, radiological, serological, and pathological data for comprehensive disease characterization. The findings of the current study, therefore, support the concept embodied in the title “Beyond Imaging,” highlighting that pathological examination contributes substantially more than diagnostic confirmation alone and provides information with potential prognostic and therapeutic significance.

The principal limitations of this study are its retrospective design, selection bias, and relatively small sample size, reflecting the inclusion of only surgically excised and histopathologically confirmed lesions. Consequently, the findings may not be representative of the entire clinical spectrum of NCC, many cases of which are successfully managed medically without tissue diagnosis. Nevertheless, this focused approach constitutes a major strength of the study, allowing direct assessment of clinicoradiological-pathological concordance and detailed evaluation of parasite morphology and host response, aspects that are often unavailable in larger imaging-based series.

Overall, the findings of the present study reinforce and expand upon existing literature regarding the pathological spectrum of NCC. Our results align with previous reports concerning demographic characteristics, inflammatory evolution, and histopathological features of NCC while extending current knowledge by providing detailed evidence of the diagnostic limitations of imaging and operative assessment in surgically excised lesions. The study underscores that, despite remarkable advances in neuroimaging, histopathological examination remains indispensable for definitive diagnosis, disease staging, and comprehensive understanding of parasite-host interactions in NCC.

## Conclusions

NCC demonstrates a broad spectrum of clinical, radiological, intraoperative, and histopathological manifestations that may closely mimic a variety of neoplastic, inflammatory, and other infectious intracranial lesions. Although modern neuroimaging techniques provide valuable diagnostic information, their accuracy may be limited in atypical or diagnostically uncertain lesions. In the present study, histopathological examination established the diagnosis in all surgically excised cases and provided important insights into parasite viability, stage of degeneration, inflammatory response, and calcification. These findings emphasize the indispensable role of histopathological evaluation as the definitive diagnostic tool in surgically excised atypical parenchymal and extraparenchymal NCC, particularly in cases where clinical and radiological features pose significant diagnostic challenges. Greater awareness of the varied histological appearances of NCC can facilitate timely diagnosis, optimize patient management, and prevent potential diagnostic errors in routine neurosurgical and neuropathological practice.

## References

[1] Hisaeda H (2026). Neurocysticercosis. [Article in Japanese]. Brain Nerve.

[2] Garcia HH, Gonzalez AE, Gilman RH (2011). Cysticercosis of the central nervous system: how should it be managed?. Curr Opin Infect Dis.

[3] Dametto E (2016). Histopathology of the human brain in neurocysticercosis. J Mol Histol Med Physiol.

[4] Del Brutto OH, Nash TE, White AC Jr (2017). Revised diagnostic criteria for neurocysticercosis. J Neurol Sci.

[5] Del Brutto OH (2022). Human neurocysticercosis: an overview. Pathogens.

[6] Bhatt AS, Mhatre R, Nadeesh BN, Mahadevan A, Yasha TC, Santosh V (2019). Nonneoplastic cystic lesions of the central nervous system-histomorphological spectrum: a study of 538 cases. J Neurosci Rural Pract.

[7] Hunter E, Cliff M, Armstrong M, Manji H, Jäger HR, Chiodini P, Brown M (2018). Active neurocysticercosis at the Hospital for Tropical Diseases, London: a clinical case series. Trans R Soc Trop Med Hyg.

[8] Milenković Z, Ignjatović A, Stalević M, Ranđelović M, Koraćević G, Mladenović S, Otašević S (2025). Lateral ventricle neurocysticercosis: a systematic review of case reports and case series. Foodborne Pathog Dis.

[9] Kumar R, Kumar Kalra S, Mahapatra AK (2008). Intraventricular neurocysticercus cyst(s) in Indian children. Pediatr Neurosurg.

[10] Garg RK, Rizvi I, Nigam H, Pandey S, Uniyal R (2025). Treatment outcome in patients with spinal neurocysticercosis: a systematic review of published cases and case series. Future Microbiol.

[11] Sharma BS, Sawarkar DP, Verma SK (2019). Endoscopic management of fourth ventricle neurocysticercosis: description of the new technique in a case series of 5 cases and review of the literature. World Neurosurg.

[12] Kotha VK (2013). Migration of intraventricular neurocysticercus after ventriculostomy. Asian J Neurosurg.

[13] Alam MR, Tripathi R, Bano H, Prithviraj M (2025). A case series on neurocysticercosis without seizures in alcohol-dependent patients. Indian J Psychiatry.

[14] Kimura-Hayama ET, Higuera JA, Corona-Cedillo R (2010). Neurocysticercosis: radiologic-pathologic correlation. Radiographics.

[15] Alsina GA, Johnson JP, McBride DQ, Rhoten PR, Mehringer CM, Stokes JK (2002). Spinal neurocysticercosis. Neurosurg Focus.

[16] Nash TE, Bartelt LA, Korpe PS, Lopes B, Houpt ER (2014). Calcified neurocysticercus, perilesional edema, and histologic inflammation. Am J Trop Med Hyg.

